# Superior Stability and Efficiency Over 20% Perovskite Solar Cells Achieved by a Novel Molecularly Engineered Rutin–AgNPs/Thiophene Copolymer

**DOI:** 10.1002/advs.201800568

**Published:** 2018-10-12

**Authors:** Ahmed Mourtada Elseman, Walid Sharmoukh, Sajid Sajid, Peng Cui, Jun Ji, Shangyi Dou, Dong Wei, Hao Huang, Wenkang Xi, Lihua Chu, Yingfeng Li, Bing Jiang, Meicheng Li

**Affiliations:** ^1^ State Key Laboratory of Alternate Electrical Power System with Renewable Energy Sources School of Renewable Energy North China Electric Power University Beijing 102206 China; ^2^ Electronic & Magnetic Materials Department Advanced Materials Division Central Metallurgical Research and Development Institute (CMRDI) Helwan P.O. Box 87 Cairo 11421 Egypt; ^3^ Department of Inorganic Chemistry National Research Centre Dokki Giza 12622 Egypt

**Keywords:** hole‐transporting materials, perovskite solar cells, silver nanoparticles, stability, thiophene

## Abstract

Perovskite solar cells (PSCs) with efficiencies greater than 20% have been realized mostly with expensive spiro‐MeOTAD hole‐transporting material. PSCs are demonstrated that achieve stabilized efficiencies exceeding 20% with straightforward low‐cost molecularly engineered copolymer poly(1‐(4‐hexylphenyl)‐2,5‐di(thiophen‐2‐yl)‐1*H*‐pyrrole) (PHPT‐py) based on Rutin–silver nanoparticles (AgNPs) as the hole extraction layer. The Rutin–AgNPs additive enables the creation of compact, highly conformal PHPT‐py layers that facilitate rapid carrier extraction and collection. The spiro‐MeOTAD‐based PSCs show comparable efficiency, although their operational stability is poor. This instability originated from potential‐induced degradation of the spiro‐MeOTAD/Au contact. The addition of conductive Rutin–AgNPs into PHPT‐py layer allows PSCs to retain >97% of their initial efficiency up to 60 d without encapsulation under relative humidity. The PHPT‐py/ Rutin–AgNPs‐based devices surpass the stability of spiro‐MeOTAD‐based PSCs and potentially reduce the fabrication cost of PSCs.

## 1 Introduction

Organometal trihalide perovskite solar cells (PSCs) have been emerged as a kind of encouraging alternatives to existing photovoltaic technologies with both solution‐processability and superior photovoltaic performance.^1^, ^2^, ^3^, ^4^, ^5^, ^6^ In 2009, Miyasaka and co‐workers reported the first overture of PSC with a power conversion efficiency (PCE) of 3.8%.^7^ Since then significant progress has been made in the PSCs performances through facile fabrication processes, compositional engineering of perovskite materials, and improved device structure.^1^, ^2^, ^3^, ^4^, ^5^, ^6^, ^8^, ^9^, ^10^ Today, the world's efficient PSC hits a 22.1% PCE, causing much excitement in the photovoltaic community.^11^, ^12^

Despite the rapid progress in terms of PCEs, the state‐of‐the‐art PSCs use expensive and unstable organic hole‐transporting materials (HTMs) such as poly[bis(4‐phenyl)(2,4,6‐trimethylphenyl)amine] (PTAA) or [2,2′,7,7′‐tetrakis(*N*,*N*‐di‐p‐methoxyphenyl‐amine)9,9′‐spirobifluorene] (spiro‐MeOTAD) that hinder long‐term stability of the devices.^13^, ^14^, ^15^, ^16^ Moreover, the cost of these HTMs is prohibitively high for practical applications, and the tedious synthetic protocols of such organic HTMs or their high purity ingredients' requirements profoundly are a factor in the commercialization of the perovskite devices that use them.^17^, ^18^ One approach to combat these issues of cost and instability could be the implementation of inexpensive thiophene‐based HTMs.^19^, ^20^, ^21^, ^22^ However, the PSCs incorporated with thiophene HTMs delivered lower PCEs compared with standard spiro‐MeOTAD.^23^ Plainly, thiophens derivatives symbolize a class of building blocks for organic semiconductor materials and have investigated to own favorable optoelectronic properties. Specially, their high hole mobility attends an attractive feature for HTM design.^24^ Recently, Grimsdale and co‐workers^25^ reported the application of small molecule based on thiophene derivatives that reveals a relatively PCE of 13.8%. From cyclic voltammetry (CV) studies and UV–vis measurements the calculated HOMO was 50 mV higher than that of spiro‐MeOTAD, which resulted in increase in open circuit volt. Also, the doping of these molecules with Co^III^ complex gave an increase in absorption.^24^ Therefore, obtaining stable PCEs over 20% with PSCs that use low‐cost synthetic protocols of thiophene‐based HTMs from commercially available and inexpensive starting materials has remained a challenge.^26^, ^27^

The development of novel small‐molecule organic semiconductors demands a better understanding of HTM structure because a proper selection of hole‐selective layer is very crucial, not only for reducing the cost, but also for obtaining high stability of the PSCs.^28^ Among various polymers, thiophene represents a class of building blocks for organic semiconductor materials owing to their favorable optoelectronic properties.^29^ In particular, their high hole mobility presents an attractive feature for HTM design.^30^ Moreover, thiophene interaction can promote photogenerated hole transport. These features inspired us to develop low‐cost and effective thiophene‐based hole‐selective layers possessing combined higher hole mobility and stability than the above‐mentioned organic HTMs.^31^

Herein, we report a facile synthesis and characterizations of novel thiophene‐based HTM as from monomers to copolymer, and its application in PSCs to obtain higher PCEs. The PSCs with synthesized thiophene derivative as HTM, and Rutin–AgNPs as additive exhibited a stable overall PCE of 21.1%, while spiro‐MeOTAD delivered a 20.8% efficiency. Meanwhile, the stability of PSCs with poly(1‐(4‐hexylphenyl)‐2,5‐di(thiophen‐2‐yl)‐1*H*‐pyrrole) (PHPT‐py)/ Rutin–AgNPs‐based devices have been enhanced dramatically, which maintains 98% of the initial efficiency even under the relative humidity at 55% without encapsulation under continuous full sun illumination at maximum power point tracking. Our work presents a simple strategy to design HTM for highly efficient and stable PSCs at low‐cost with commercial viability.

## 2 Results and Discussions

**Figure 1:**
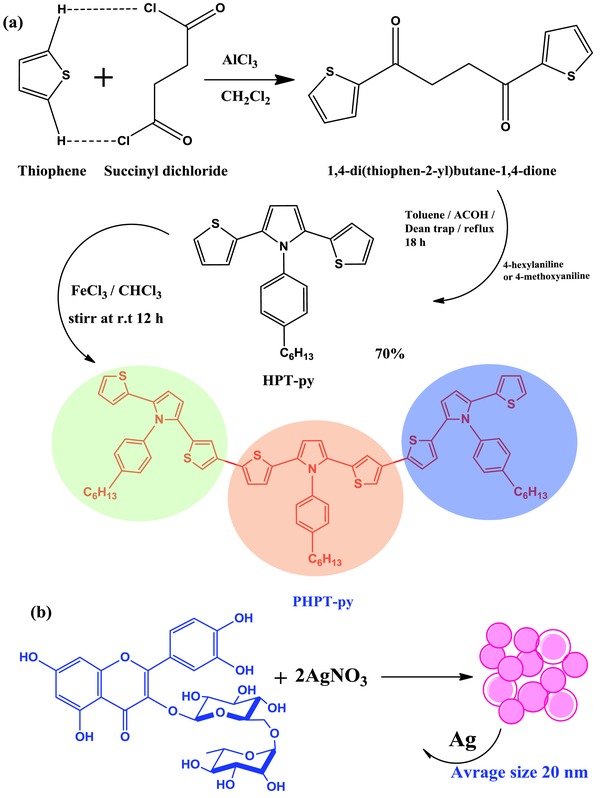
The molecular structures and synthetic route for a) PHPT‐py as HTM and b) additives materials from Rutin and silver nitrate.

The detail synthetic route and the chemical reaction for the HTM were presented in **Figure** **1** and Supporting Information. The monomer HPT‐py was prepared by palladium catalyst C—C bond and potassium acetate through Suzuki coupling reaction^32^ of 2‐bromothiophene to obtain a pure compound in 80% yield.^33^ The monomer was carried out under oxidative coupling conditions for polymerizations according to literatures.^34^, ^35^ This new thiophene PHPT‐py derivatives was fully characterized by ^1^H NMR and ^13^C NMR spectroscopy. All the analytical data were consistent with the proposed structures (see Figures S1 and S2, Supporting Information). We also roughly estimated the synthesis cost of 1 g (PHPT‐py) in comparison to common HTMs in PSCs. The details were shown in Table S1 in the Supporting Information The estimated synthesis cost of PHPT‐py is 49 $ g^−1^ which are comparably cheaper than spiro‐MeOTAD (603.65 $ g^−1^). Also, we used Rutin–AgNPs as an additive material to improve the Ohmic contact between HTM and perovskite layer. Rutin as metal chelation belong to flavonoids, which are polyphenol compounds widely distributed in plants.^36^, ^37^ In this work, the strategy of silver chelation on Rutin was proposed for increasing the hole conductivity in PHPT‐py HTM. The Rutin–AgNPs was fully characterized by ^1^H NMR and ^13^C NMR spectroscopy. All the analytical data were consistent with the proposed structures. The surface morphology (SEM), chemical compositional analyses, and transmission electron microscope (TEM) of the synthesized Rutin–AgNPs were shown in Figure S3 in the Supporting Information. The morphology revealed a spherical structure for silver surrounded by Rutin molecules. The particle size was averaged between 19 and 40 nm for the whole particles. The EDX spectra of AgNPs confirmed the presence of 90.3%, 5.7%, and 4% for Ag, Cl, and C elements, respectively.

**Figure 2:**
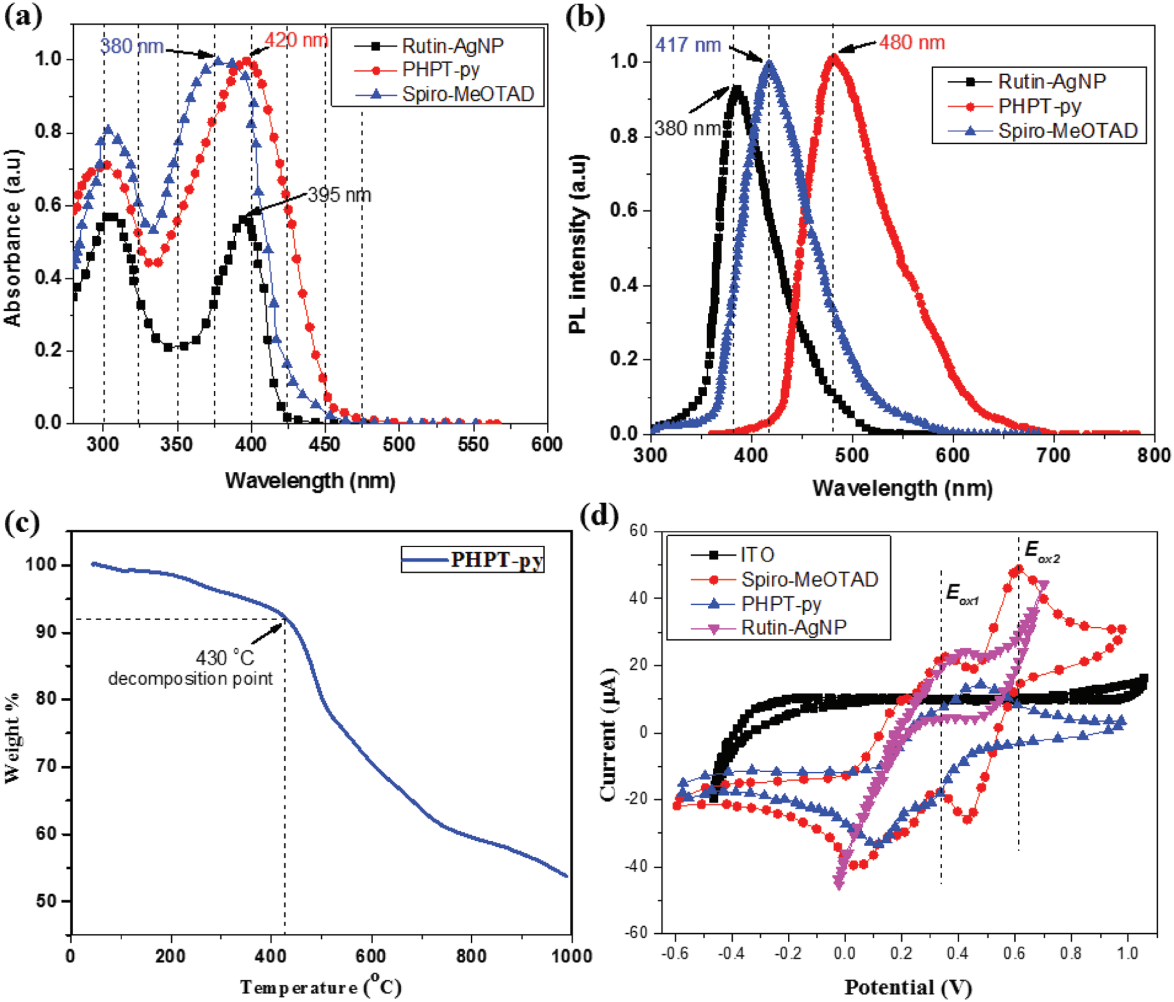
a) Normalized UV–vis absorption spectra of the HTMs in acetonitrile solution. b) Normalized PL spectra of HTMs thin film. c) TGA curves of the PHPT‐py. d) CV curves of HTMs in dichloromethane solution.

The electronic properties of the new Rutin–AgNPs, PHPT‐py, and spiro‐MeOTAD were investigated by UV–vis absorption and fluorescence emission spectroscopy as shown in **Figure** **2**a,b. Figure 2a presents the UV–vis absorption spectra of Rutin–AgNPs, PHPT‐py, and spiro‐MeOTAD in acetonitrile solution while the related numerical data of absorption and electrochemical properties were illustrated in Table S2 in the Supporting Information. According to our illustrated results, the PHPT‐py showed different major absorption bands (300–460 nm) which can be attributed to the localized aromatic π–π* or n–π* transitions of the major conjugated structure. The absorption bands in the 300–320 nm region can be assigned to the n–π* transition of the hexylthiophene moieties.^38^ The maximum absorption of PHPT‐py (420 nm) shows considerable red‐shift which can be ascribed to the positive effect of the hexyl chain compared with spiro‐MeOTAD. The absorptions at 420 and 380 nm of PHPT‐py and spiro‐MeOTAD in solution were attributed to the π–π* transition of conjugated system unit and thiophene ring. While, the absorption at 395 nm of Rutin–AgNPs in solution was attributed to the π–π* transition of a larger conjugated system formed by bridging the triphenyl phenol unit. Due to the larger degree of conjugation produced by introducing two double bonds, the λ_abs/max_ of PHPT‐py shows a red‐shift compared with spiro‐MeOTAD. The optical energy bandgaps (*E*_g_) of Rutin–AgNPs, PHPT‐py, and spiro‐MeOTAD were estimated from the onset of the absorption spectra which were determined to be 3.14, 2.95, and 3.01 eV, respectively. The photoluminescence (PL) spectra of the Rutin–AgNPs, PHPT‐py, and spiro‐MeOTAD thin films were displayed in Figure 2b. The PL measurements were carried out in thin film state on photo excitation at 465 nm. The HTMs of PHPT‐py and spiro‐MeOTAD exhibited blue and violet emission peaks at around 480 and 417 nm, respectively. The Rutin–AgNPs shows near‐ultraviolet emission peak at around 380 nm. The high PL efficiency was due to the sterically hindered hexyl group in the phenyl ring, which inhibits the intermolecular interaction between the polymer backbones. Thermogravimetric analysis (TGA) measurement showed that the PHPT‐py HTM has high decomposition temperatures *T*_d_ = 430 °C and glass transition temperatures *T*_g_ = 85 °C (Figure 2c). We performed CV measurement to determine their energy levels experimentally as shown in Figure 2d. The data were summarized in Table S2 in the Supporting Information. For better comparison of energy levels, we measured the cyclic voltammogram of the HTMs under the same condition with ferrocene as internal standard (see Figure S4, Supporting Information). According to the onset potential of the reduction and oxidation process, the lowest unoccupied molecular orbital (LUMO) and highest occupied molecular orbital (HOMO) energy levels of polymers can be calculated using Equations (1) and (2):

(1) $$E_{\mathrm{HOMO}}=-\left(E_{\left[\mathrm{onset},\;\mathrm{ox}\right]}+4.4\right)\left(\mathrm{eV}\right)$$

(2) $$E_{\mathrm{LUMO}}=-\left(E_{\left[\mathrm{onset},\;\mathrm{red}\right]}+4.4\right)\left(\mathrm{eV}\right)$$

For the favorable hole transfer process from the perovskite layer the HOMO energy levels of Rutin–AgNPs, PHPT‐py, and spiro‐MeOTAD are − 4.97, − 5.09, and − 5.12 eV, respectively, which are slightly lower than that of spiro‐MeOTAD. The LUMOs of HTMs were calculated to be − 1.83, − 2.14, and − 2.11 eV, which are more positive than that of CH_3_NH_3_PbI_3_ (− 3.9 eV). These results agreed well with the trend derived from DFT calculations which were compared in Table S2 in the Supporting Information.

**Figure 3:**
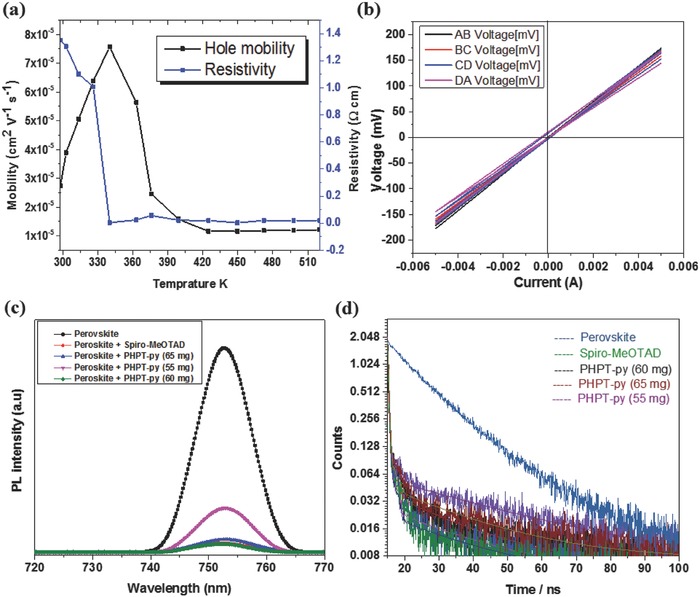
a) Charge mobility and resistivity as a function of temperature of the PHPT‐py thin film. b) *I*–*V* characteristics (Ohmic behavior) for of HTMs thin film. c,d) PL spectra and TRPL spectra of corresponding films on glass substrate.

Furthermore, we carried out the charge carrier mobility and resistivity of Rutin–AgNPs/ PHPT‐py thin films by Hall‐effect measurement. **Figure** **3**a shows hole mobility (μ_h_) as a function of temperature. It can be seen that the μ_h_ of the prepared Rutin–AgNPs/ PHPT‐py was increased gradually and become maximum at 340 K. For instance, the hole mobility was recorded 7.6 × 10^−5^ cm^2^ V^−1^ s^−1^ at 340 K.^8^ Then, this value was decreased with increasing temperature. We conclude that the generation of hole–electron pairs depends on the suitable temperature. On the other hand, the initial resistivity of 1.30 × 10^3^ Ω cm was recorded at 295 K as depicted in Figure 3a. The relationship between temperature and resistivity of the Rutin–AgNPs/PHPT‐py film described that the resistivity was decreased with increasing temperature. The change in conductivity was attributed to the movement of electron–hole pairs because at high temperature the covalent bonds were broken and as a result more electron–hole pairs were available for conduction. In other words, with increasing temperature, the electrons from valence band acquired energy and jumped to conduction band. This indicated the continuation of reduce resistance with increasing charge carrier concentration in the conduction band. The positive sign of Hall coefficient in Hall‐effect measurement showed the similar p‐type semiconductor's behavior. Moreover, the current–voltage (*I*–*V*) curves between each pair of contacts AB, BC, CD, and DA for the Rutin–AgNPs/ PHPT‐py thin film at different temperatures between 295 and 522 K were shown in Figure 3b. In all cases, the linearity of *I*–*V* curves ascribed suitable Ohmic contacts.^20^, ^39^, ^40^

The charge extraction phenomena were investigated by steady‐state PL spectra as shown in Figure 3c. The strong PL quenching was observed when the HTMs were coated on perovskite films. Herein, three different concentrations of 65, 60, and 55 mg from Rutin–AgNPs/PHPT‐py (HTM) and spiro‐MeOTAD were coated over the perovskite films for the optimization of PL quenching. The PL intensity was reduced to 7%, 3%, and 15% for Rutin–AgNPs/PHPT‐py and 3.2% for Spiro‐MeOTAD compared with pristine films. These results suggesting that fast interface charge separation in Rutin–AgNPs/PHPT‐py (60 mg)‐based devices can contribute to a better short‐circuit current density (*J*_SC_) and FF in the PSCs compared with other concertation. Plainly, the PL results were similar for Rutin–AgNPs/PHPT‐py (60 mg) and spiro‐MeOTAD‐based perovskite devices. The hole extraction capabilities at glass/perovskite/HTMs interfaces were investigated by time‐resolved photoluminescence (TRPL) measurement (Figure 3d). The corresponding decay time was obtained by fitting the data with biexponential decay function. In biexponential PL decay process, the short‐lived lifetime (τ_1_) correlates with surface property and/or nonradiative recombination and the long‐lived lifetime (τ_2_) relates to the bulk property. The pristine perovskite film displayed τ_1_ = 15.0 ns and τ_2_ = 185.80 ns. The Rutin–AgNPs/PHPT‐py (65, 60, and 55 mg)/perovskite and spiro‐MeOTAD/perovskite films showed short‐lived and long‐lived lifetimes of ( τ_1_ = 0.5 ns, τ_2_ = 1.6 ns), (τ_1_ = 0.4 ns, τ_2_ = 1.0 ns), ( τ_1_ = 0.7 ns, τ_2_ = 2.3 ns), and (τ_1_ = 0.3 ns, τ_2_ = 0.8 ns), respectively. The PL decay lifetime for these devices were significantly shorter than perovskite layer without HTM layers. It was confirmed by PL quenching and TRPL that both Rutin–AgNPs/PHPT‐py (60 mg) and spiro‐MeOTAD extract holes efficiently from perovskite.

**Figure 4:**
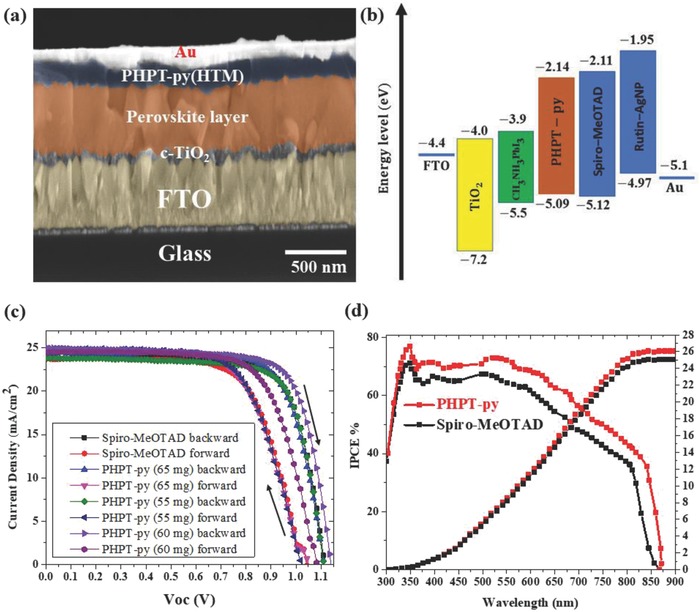
a) Cross‐sectional SEM image of the device based on PHPT‐py. b) Energy band level diagram of the corresponding materials used in PSCs. c) Current–voltage hysteresis curves of PSCs comprising champion devices measured starting with backward scan and continuing with forward scan. d) Internal quantum efficiency (IQE) spectra and integrated current curves of the corresponding devices.

In this paper, we reported the development of a new silver complex with Rutin (Rutin–AgNP). The structure and synthetic mechanism were explained in Figure 1b. More details about the synthetic procedures and the microstructure characterization were described in the Supporting Information. The Rutin–AgNP in presence of 4‐tert‐butylpyridine (TBP), lithium bis(trifluoromethylsulphonyl)imide (Li‐TFSI), and *tris*[2‐(1*H*‐pyrazol‐1‐yl)‐4‐tertbutylpyridine)]cobalt(III) *tris*(bis(trifluoromethylsulfonyl)imide) (Co(PyPyz)_3_[TFSI]_3_), were added to PHPT‐py to further improve the properties of the HTM layer and consequently the overall cell performance. The Rutin–AgNPs/PHPT‐py (65, 60, and 55 mg) and Spiro‐MeOTAD HTMs were spin‐coated in the device architecture of fluorine‐doped tin oxide (FTO)/TiO_2_(compact)/perovskite/HTM/Au, using the similar method as reported in our recent paper.^41^ The cross‐section image of the PSC shown ≈100 nm compact TiO_2_, ≈ 460 nm perovskite, ≈ 120 nm HTM layer, and 80 nm thick gold back contact as analyzed by field‐emission scanning electron microscopes (FE‐SEM) in **Figure** **4**a.

The current–voltage (—*J*–*V*) characteristic curves were collected under simulated solar illumination (AM 1.5, 100 mW cm^−2^) for the PSCs incorporated with Rutin–AgNPs/PHPT‐py (65, 60, and 55 mg) and spiro‐MeOTAD HTMs as illustrated in Figure 4c. The corresponding data of photovoltaic parameters were summarized in **Table** **1**. The best device based on Rutin–AgNPs/PHPT‐py (60 mg) exhibited an open‐circuit voltage (*V*_oc_) of 1.109 V, a *J*_SC_ of 24.94 mA cm^−2^ and a fill factor (FF) of 0.76 and a PCE of 21.1%. The other different concentration from Rutin–AgNPs/PHPT‐py (65 and 55 mg) showed an *V*_oc_ of 1.11 and 1.10 V, *J*_SC_ of 22.96 and 21.07 mA cm^−2^ and FF of 0.74 and 0.74, leading to a PCE of 18.7% and 17.2%, respectively. In comparison, the PSCs based on spiro‐MeOTAD delivered a *J*_SC_ of 25.25 mA cm^−2^, *V*_oc_ of 1.11 V, and FF of 0.74 and PCE of 20.78%. Moreover, the hysteresis of PSCs with Rutin–AgNPs/PHPT‐py was also reduced. The low hysteresis of the PHPT‐py‐based PSCs could be originated to the improved interconnectivity between perovskite and Rutin–AgNPs/PHPT‐py. These improvements were further attributed to the lower interfacial defect density, reduced trap states and enhanced interfacial charge transfer. On the other hand, the incident‐photon‐to‐current‐efficiency (IPCE) spectra of the PSCs were presented in Figure 4d. It can be seen that the generation of photocurrent began at 850 nm, which was consistent with the bandgap of perovskite, and attained peak values greater than 80% in the short‐wavelength region of the visible spectra. Moreover, the similar shape of IPCE spectra for all devices indicated that light absorption by the HTM is negligible and the role of these compounds is limited to hole transporting only. The integrated current densities calculated from the IPCE spectra were 25.26 and 25.9 mA cm^−2^ for PCSs based on Rutin–AgNPs/PHPT‐py (60 mg) and spiro‐MeOTAD, respectively. These results are in good agreement with the current densities obtained from the *J*–*V* curves.

**Table 1:** Champion devices *J*–*V* curves under different scan directions

HTM		*J*_SC_ [mA cm^−2^]	*V*_OC_ [V]	FF	PCE [%]
PHPT‐py (60 mg)	backward	24.94	1.11	76.32	21.10
	forward	24.86	1.04	68.48	17.77
PHPT‐py (65 mg)	backward	22.96	1.11	73.56	18.73
	forward	23.46	1.06	65.15	16.16
PHPT‐py (55 mg)	backward	21.07	1.10	74.46	17.20
	forward	20.91	1.03	65.31	14.08
Spiro‐MeOTAD	backward	25.25	1.11	74.05	20.78
	forward	23.85	1.06	69.13	17.43

In addition to considerable improvement in efficiency, the stability of PSCs is equally important. It is widely reported that the stability issues of PSCs mainly result from the ambient environment, and from layers adjacent to the perovskite. In this regard, we tested the stability of our PSCs without encapsulation for 60 d under AM1.5 illumination condition. In the measurement intervals, the PSCs were taken out for the testing at ambient environment and then stored in the glovebox for the next test. The PSCs with Rutin–AgNPs/PHPT‐py exhibited an excellent long‐term stability compared with the standard spiro‐MeOTAD‐based PSCs as shown in Figure S5 in the Supporting Information. After the continuous operational measurement for 2 months, the PCE of PSCs with Rutin–AgNPs/PHPT‐py changed slightly from 21.1% to 19.23%, maintaining 98% of the initial efficiency. In contrast, the PCE of the PSCs based on spiro‐MeOTAD decreased severely from 20.8% to 17.1%, indicating the poor stability and rapid degradation under testing conditions.

The stability tests of corresponding PSCs at ambient environment of 55% relative humidity and under continuous full sun illumination at maximum power point tracking at room temperature without encapsulation shown in Figure S6a in the Supporting Information. Obviously, the devices based on Rutin–AgNPs/PHPT‐py present a better stability than that of spiro‐MeOTAD. As shown in Figure S6a in the Supporting Information, the PCE maintained 86% of the initial value in the PSC based on Rutin–AgNPs/PHPT‐py after 500 h, whereas it almost decreased of the initial value in spiro‐MeOTAD. Moreover, preliminary tests in Figure S6a in the Supporting Information show that PHPT‐py‐based device is more resistant to humidity and heat stress than the device based on spiro‐MeOTAD. There is no doubt that the difference of the stability is related to interaction and improved morphology and hydrophobicity of the HTM by the new additives and the HTM/perovskite interface. Furthermore, the aging operation of tracking the maximum power point of the PSCs with Rutin–AgNPs/PHPT‐py and spiro‐MeOTAD was also performed as shown in Figure S6b in the Supporting Information. All the PSCs were measured by tracking the maximum power point of 0.95 V at room temperature under the AM 1.5G illumination of Xe‐lamp without any filter in ambient air. In Figure S6b in the Supporting Information, it is obvious that the PSCs with Rutin–AgNPs/PHPT‐py exhibit more stable performance than the PSCs with spiro‐MeOTAD after a continuous illumination for 2000 s. The PSCs‐based PHPT‐py maintain 88% of the initial efficiency, in contrast, the efficiency of PSCs‐based spiro‐MeOTAD almost degraded.

The structure of triphenyl with amine and sulfur groups can effectively prevent the π accumulation, inhibiting the occurrence of crystallization and enhancing the geometrical structure with electron cycling. It can also reduce the direct contact between Au electrode and the light absorbing layer, and thus effectively block the hole and electron recombination. Furthermore, the introduction of hexyl group can improve the solubility of the material. Moreover, the conjugation effect of the PHPT‐py molecule contributes to the efficient holes transmission. On the other hand, Rutin–AgNP playing an important role in the efficiency of PHPT‐py as HTM. The mechanism of PHPT‐py interaction with the Rutin–AgNPs can be explained from two points. First, the Rutin–AgNPs was oxidized at the cathode surface. Then, the PHPT‐py attracted to the Au surface and subsequently reacts with the oxidized form of Rutin. Thus, PHPT‐py is more oxidized and Rutin is deoxidized‐matching reaction. Second, the hexyl and thiophene function group of PHPT‐py attracted perovskite surface. Consequently, the effect of Rutin with AgNPs resulting in the increase of concentration and out‐crust catalysis. Then, PHPT‐py displays significant oxidation response due to matching reaction in first step with probably contributed cooperation of PHPT‐py and Rutin–AgNPs. Also, the new type chelation‐like silver metal complex interaction indicates that the introduced Rutin–AgNPs can restrict the migration of halides group in perovskite through the chelation interaction between Ag and I with the geometry of Rutin structure.

## 3 Conclusion

In summary, the synthesized novel thiophene‐based HTM (PHPT‐py) dopant with Rutin–AgNPs was applied as hole‐transporting layer in planar PSCs. The insertion of hexyl group into PHPT‐py gave facile synthesis, low‐cost, excellent performance, and a simple strategy for potential HTM design in highly efficient and stable PSCs. The new additive Rutin–AgNPs used in HTM presents a more homogeneous surface, higher hole mobility, and efficient charge extraction. The Rutin–AgNPs/PHPT‐py‐based PSC exhibits a PCE of 21.1%, which is superior to the conventional spiro‐MeOTAD. The devices based on Rutin–AgNPs/PHPT‐py also obtained a higher stability than that of spiro‐MeOTAD at room temperature without encapsulation. This work demonstrates that Rutin–AgNPs/PHPT‐py is a very promising hole‐transporting layer for fabricating efficient and stable PSCs.

## 4 Experimental Section

*Device Fabrication*: FTO‐coated glass substrates were cleaned by ultrasonication in an alkaline, aqueous washing solution, rinsed with deionized water, ethanol, acetone, and subjected to an O_3_/ultraviolet treatment for 30 min. After cleaning the substrates, an aqueous stock solution of 2 M TiCl_4_ (stored in the freezer) was diluted to the required concentration.^41^, ^42^ The electrodes with the tapes were then immersed into this solution and kept in an oven at 70 °C for 1 h in a closed vessel. After 1 h the electrodes were washed with deionized water and ethanol, and then dried at 100 °C in air for an hour to obtain compact‐TiO_2_ layer. The CH_3_NH_3_PbI_3_ films were fabricated using a modified one‐step solution deposition method with consecutive diethyl ether dripping. The perovskite precursor solution contained 159 mg of CH_3_NH_3_I, and 461 mg of PbI_2_ in anhydrous DMF/DMSO (600 µL/78 µL) solution, which was spin‐coated directly on the ultraviolet–ozone‐treated FTO substrate at 4000 r.p.m. for 30 s. During the spinning step, 0.5 mL of diethyl ether was slowly poured on the spinning substrate 20 s before the end. The substrates were then annealed at 130 °C for 10 min in a nitrogen‐filled glovebox. The HTM was then deposited by spin coating at 4000 r.p.m. for 30 s. The spin‐coating formulation was prepared by dissolving three different concentrations from PHPT‐py (65, 60, and 55 mg) in chlorobenzene. Each concentration of PHPT‐py was dissolved with Rutin–AgNP (see the Supporting Information), 28.8 µL TBP, 17.5 µL of a stock solution of 520 mg ml^−1^ Li‐TFSI in acetonitrile, and 29 µL of a stock solution of 300 mg mL^−1^ Co(PyPyz)_3_[TFSI]_3_ in acetonitrile in 1 mL chlorobenzene. The same method was repeated with 72.3 mg from spiro‐MeOTAD except Rutin–AgNPs. Finally, 80 nm of gold was thermally evaporated on top of the HTM layers to form the back contact. The device fabrication was carried out under controlled atmospheric conditions and humidity of less than 1%.

*Device Characterization*: Transient PL decay of the perovskite films on glass substrates was measured using a transient state spectrophotometer (Edinburgh Ins. FL920) under the irradiation of a 635 nm pulse laser with excitation energy around 3 nJ cm^−2^. Laser confocal microscope measurement of the perovskite films on glass substrates were conducted using Leica TCS‐SP5 with a 532 nm laser. Current–voltage curves were measured using a source meter (Keithley 2400) under AM 1.5G irradiation with a power density of 100 mW cm^−2^ from a solar simulator (XES‐301S+EL‐100).

## Conflict of Interest

The authors declare no conflict of interest.

## Supporting information

Supplementary
