# Supplementary material for: Superior Stability and Efficiency Over 20% Perovskite Solar Cells Achieved by a Novel Molecularly Engineered Rutin–AgNPs/Thiophene Copolymer
Source: Adv Sci (Weinh). 2018 Oct 12;5(11):1800568. doi: 10.1002/advs.201800568 (PMC6247057; doi:10.1002/advs.201800568)
Supplement: Supplementary file 1 — Supplementary [file ADVS-5-1800568-s001.pdf]

Copyright WILEY-VCH Verlag GmbH & Co. KGaA, 69469 Weinheim, Germany, 2018.

ADVANCED SCIENCE
Open Access

Supporting Information

for *Adv. Sci.,* DOI: 10.1002/advs.201800568

Superior Stability and Efficiency Over 20% Perovskite Solar Cells Achieved by a Novel Molecularly Engineered Rutin–AgNPs/Thiophene Copolymer

*Ahmed Mourtada Elseman, Walid Sharmoukh, Sajid Sajid, Peng Cui, Jun Ji, Shangyi Dou, Dong Wei, Hao Huang, Wenkang Xi, Lihua Chu, Yingfeng Li, Bing Jiang, and Meicheng Li**

Copyright WILEY-VCH Verlag GmbH & Co. KGaA, 69469 Weinheim, Germany, 2016.

Supplementary file

Superior stability and efficiency over 20% perovskite solar cells achieved by a novel molecularly engineered Rutin-AgNPs/thiophene copolymer

*Ahmed Mourtada Elseman, Walid Sharmoukh, Sajid Sajid, Peng Cui, Jun Ji, Shangyi Dou, Dong Wei, Hao Huang, Wenkang Xi, Lihua Chu, Yingfeng Li, Bing Jiang, Meicheng Li**

Ahmed Mourtada Elseman, Sajid Sajid, Peng Cui, Jun Ji, Shangyi Dou, Dong Wei, Hao Huang, Wenkang Xi, Lihua Chu, Yingfeng Li, Bing Jiang, Meicheng Li, *
State Key Laboratory of Alternate Electrical Power, System with Renewable Energy Sources, School of Renewable Energy, North China Electric Power University, Beijing 102206, China. E-mail: mcli@ncepu.edu.cn

Dr. Ahmed Mourtada Elseman
Electronic & Magnetic Materials Department, Advanced Materials Division, Central Metallurgical Research and Development Institute (CMRDI), Helwan, P.O. Box 87, Cairo 11421, Egypt.

Dr. Walid Sharmoukh
Department of Inorganic Chemistry, National Research Centre, Dokki, Giza 12622, Egypt.

*Corresponding author information: E-mail: mcli@ncepu.edu.cn; Tel: +86 10 6177 2951

1. Experimental

1.1Materials

All materials were available commercially and used without further purification if not mentioned specially. The chemicals and solvents were purchased from Sigma-Aldrich, Acros, Combi-Blocks America and Fisher Scientific and used as received.

1.2 Synthesis of 1,4-di(thiophen-2-yl)butane-1,4-dione

1,4-di(thiophen-2-yl)butane-1,4-dione was synthesized according to literature [1, 2]. A solution of thiophen (50.1 ml, 1.2 mol) with succinyl chloride (20.3 ml, 0.5 mol) was mixed in methylene chloride with stirring in presence of Aluminum chloride over 1 hr. after long stirring the materials were separated and washed by 2N HCl, H_2O and dried over $MgSO_4$. The product suspended in ethanol to give 1,4-di(thiophen-2-yl)butane-1,4-dione **(1)** with mp: 127-128. ^{1}H NMR (400 MHz, $CDCl_3$) δ 7.84 (d, J = 3.5 Hz, 2H), 7.67 (d, J = 4.9 Hz, 2H), 7.21 2 (t, J = 4.4 Hz, 2H), 3.43 (s, 4H) ppm.

1.3 Synthesis of 1-(4-hexylphenyl)-2,5-di(thiophen-2-yl)-1H-pyrrole (HPT-py)

The monomer was prepared through 1,4-di(thiophen-2-yl)butane-1,4-dione (5.0g, 20mmol), and 4-hexylaniline (15.2 mL, 80mmol) and then dissolved in 40 mL of a toluene-acetic acid (3:1) mixture. The mixture was refluxed with a dean stark condenser for 18 h under nitrogen, cooled to room temperature and then 100 mL of toluene was added to this mixture. The insoluble material was filtered. The obtained toluene solution was concentrated in vacuo and then poured into a saturated Na_2SO_4 Solution to make the reaction mixture basic. The organic layer was separated and then the aqueous layer was extracted with toluene (150 mL). The combined organic layers were washed with water, dried over anhydrous Na_2SO_4 and then concentrated in vacuo. The residue was purified by column chromatography (silica gel, toluene) and then recrystallized from acetone to afford the pure product. Yield: 4.5g (58%). mp 110–111 C; 1 H NMR (500 MHz, $CDCl_3$): d (ppm) 7.25 (s, 4 H), 7.02–705 (dd, 2 H), 6.79–6.83 (m, 2 H), 6.50– 6.55 (m, 4H), 2.67–2.72 (t, 2 H), 1.64–1.70 (m, 2 H), 1.29– 1.33 (m, 10 H), 0.88–0.92 (t, 3 H). 13C NMR (75 MHz, CDCl3); d (ppm) 144.50, 136.11, 135.35, 130.43, 129.93, 129.42, 127.07, 124.20, 124.01, 109.75, 35.87, 32.10, 31.48, 29.68, 29.55, 29.33, 22.93, 14.37; HRMS (EIþ, m/z) [Mþ] Calcd for C26H29NS2 419.1741, found 419.1740; Anal. Calcd for C26H29NS2: C, 74.41; H, 6.97; N, 3.34; S, 15.28. Found: C, 74.55; H, 7.00; N, 3.27; S, 15.44.

1.4 Synthesis of Poly(1-(4-hexylphenyl)-2,5-di(thiophen-2-yl)-1H-pyrrole) (PHPT-py)

The monomers were carried out under oxidative coupling conditions for polymerizations, Anhydrous $FeCl_3$ (0.65 g, 4.0 mmol) and $CHCl_3$ (1 mL) were placed in a 25-mL two-necked round-bottom flask. The flask was then filled with nitrogen, and HPT-py (0.17 g, 1.0 mmol) was added to the suspension. Three equivalents of anhydrous $FeCl_3$ (III) in chloroform at room temperature for 24 h. The reaction mixture was heterogeneous due to the insolubility of ferric chloride. After that the black or brown suspension was poured into methanolic HCl (10 wt %) and the black precipitate was filtrated off. The resulting polymers were washed with methanol then extracted with $CHCl_3$ for 24 h using a Sohxlet's extraction apparatus. The chloroform solution was removed and the title polymers products were obtained as a dark powder. The polymers yield was 60%.

1.5 Synthesis of Rutin-AgNP as additive

Nanoparticles were synthesized according to previously reported method of Rutin using plant extract with a little modification. One equivalent concentrations of Rutin and two equivalent of silver nitrate were dissolved in methanol and water. The silver nitrate solution was added to Rutin solution slowly. After that the mixture was ultrasonic cleaner for 15 min and left in dark for one day. The precipitated AgNPs were centrifuged and washed with water and methanol several times. The AgNPs were dried using freeze dryer equipment. ^{1}H and ^{13}C NMR spectra were recorded on Bruker 500 MHz instruments by using the residual signals for $CDCl_3$ at δ = 7.26 ppm and 77.0 ppm, for DMSO- d_6 at δ = 2.50 ppm and 39.4 ppm and for acetone- d_6 at δ = 2.05ppm, 29.84 ppm, and 206.26 ppm, as internal references for ^{1}H and ^{13}C respectively.

1.6 Structural, optical and electrochemical characterization

^{1}H and ^{13}C NMR spectra were recorded on Bruker 500 MHz instruments by using the residual signals for $CDCl_3$ at δ = 7.26 ppm and 77.0 ppm, for DMSO- d_6 at δ = 2.50 ppm and 39.4 ppm and for acetone- d_6 at δ = 2.05ppm, 29.84 ppm, and 206.26 ppm, as internal references for ^{1}H and ^{13}C, respectively. The UV-Vis electronic spectra were recorded on a PerkinElmer Lambda 750 UV/Vis spectrophotometer. The emission spectra were obtained using Jasco FP-6500, Japan spectrofluorometer. All electrochemical measurements were carried out in a three-electrode system using Autolab potentiostat/galvanostat PGSTAT302N, employing platinum electrode as a working electrode, platinum wire as a counter electrode, and an Ag/AgCl electrode as the reference electrode. The system was initially calibrated with

ferrocene redox couple and converted to normal hydrogen electrode (NHE). The Redox potential of HTMs study was carried out in 0.1 M tetrabutylammonium hexafluorophosphate ($TBAPF_6$) in Acetonitrile solution as the supporting electrolyte at a scan rate of 100 mV s^{-1}. TGA were carried out from room temperature to 1000 °C with a heating rate of 10.0 °C min-1 in a N_2 atmosphere on a thermogravimetric analyzer (SDT Q600).

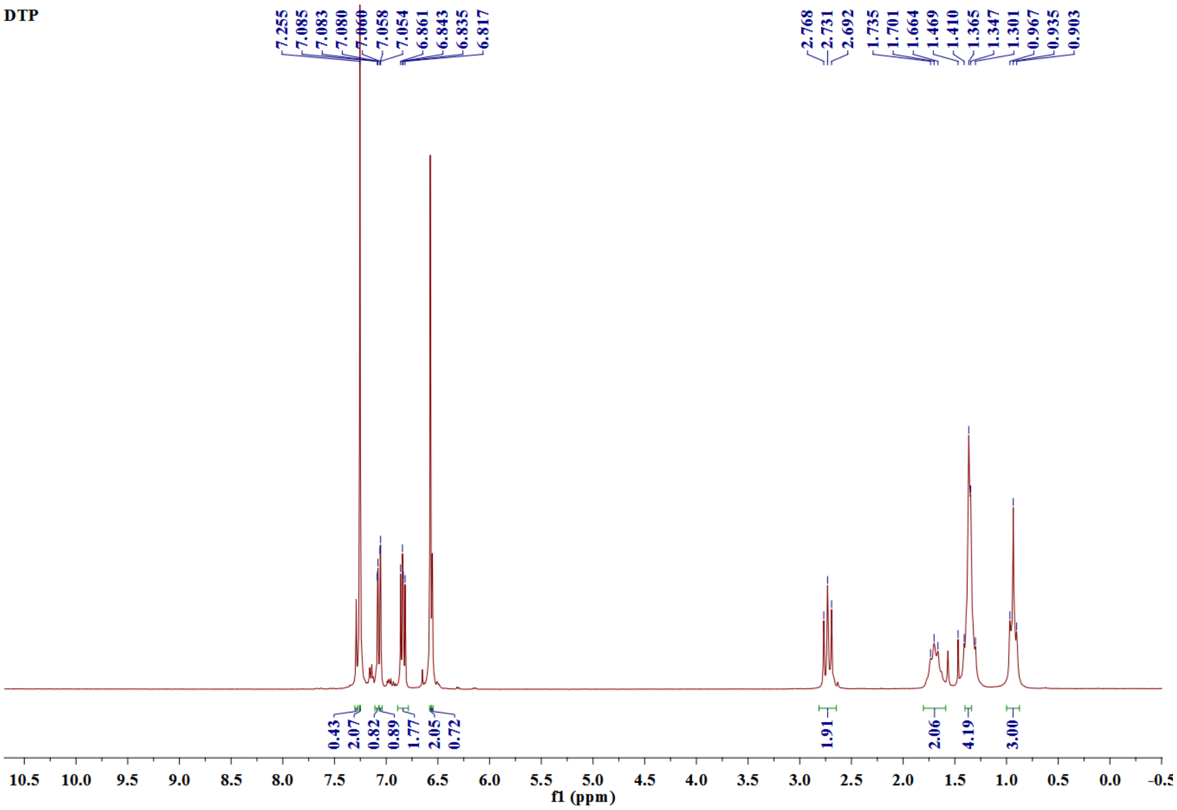

Figure S1. ^{1}H NMR spectrum of PHPT-py

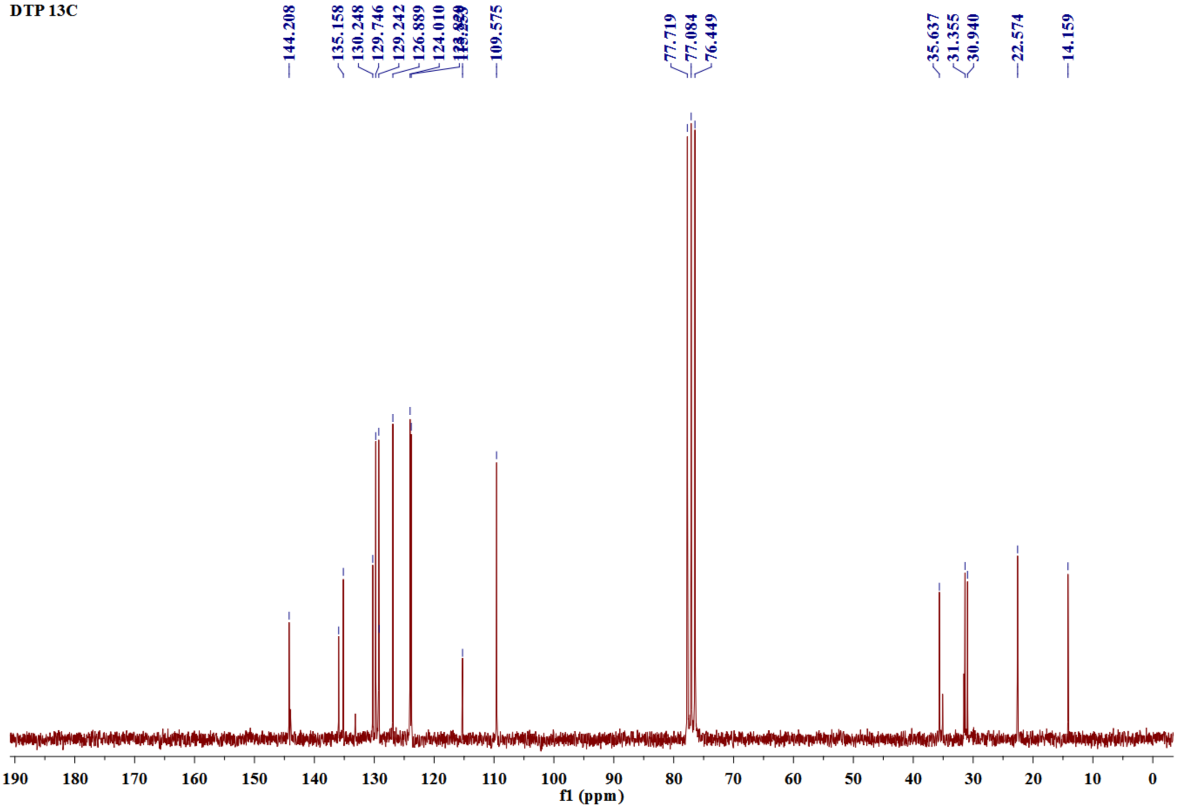

Figure S2. ^{13}C NMR spectrum of PHPT-py

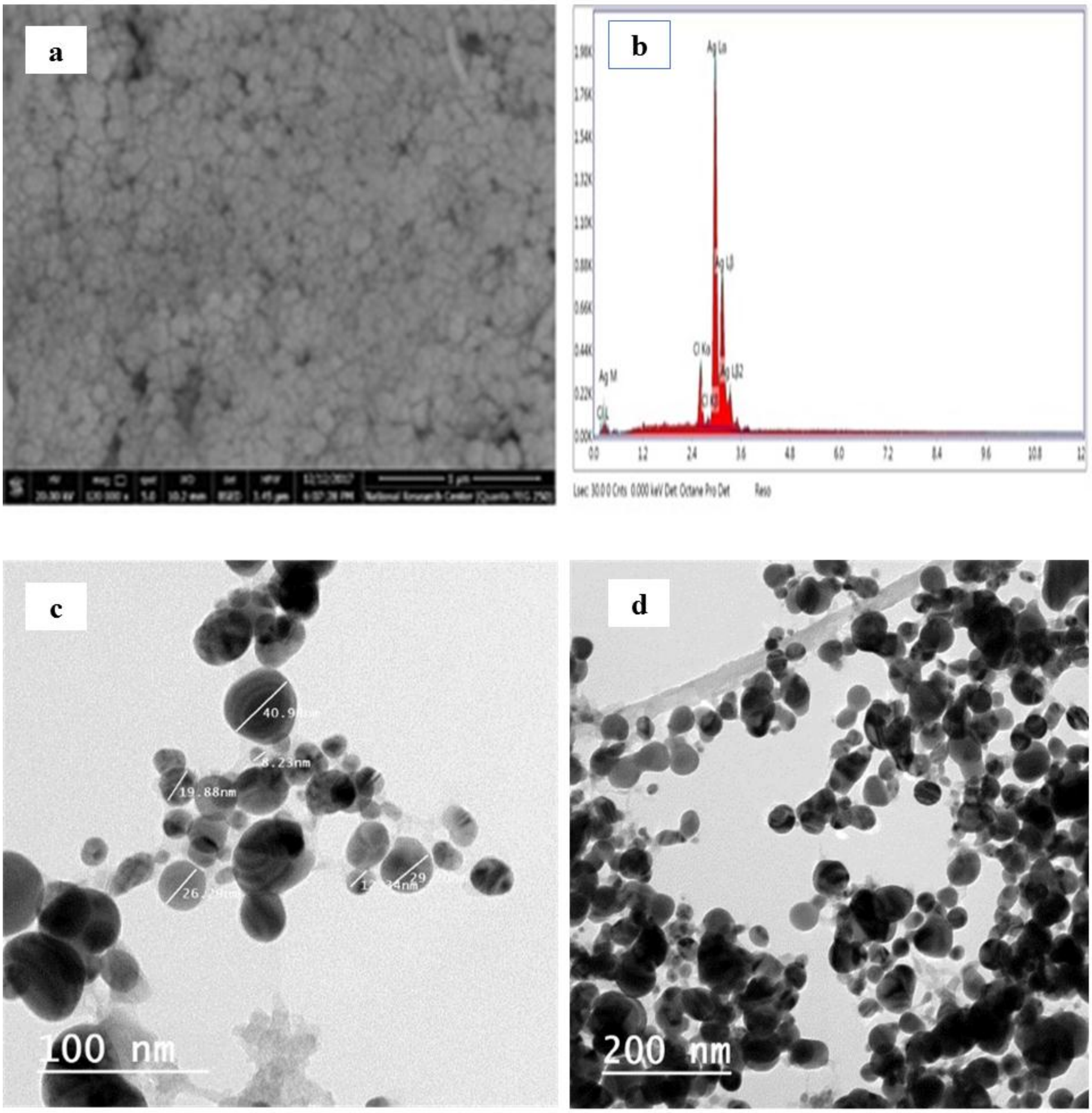

Figure S3. (a) SEM image and (b) Energy Dispersive X-ray spectrum (EDX) (c) and (d) TEM images of Rutin-AgNP.

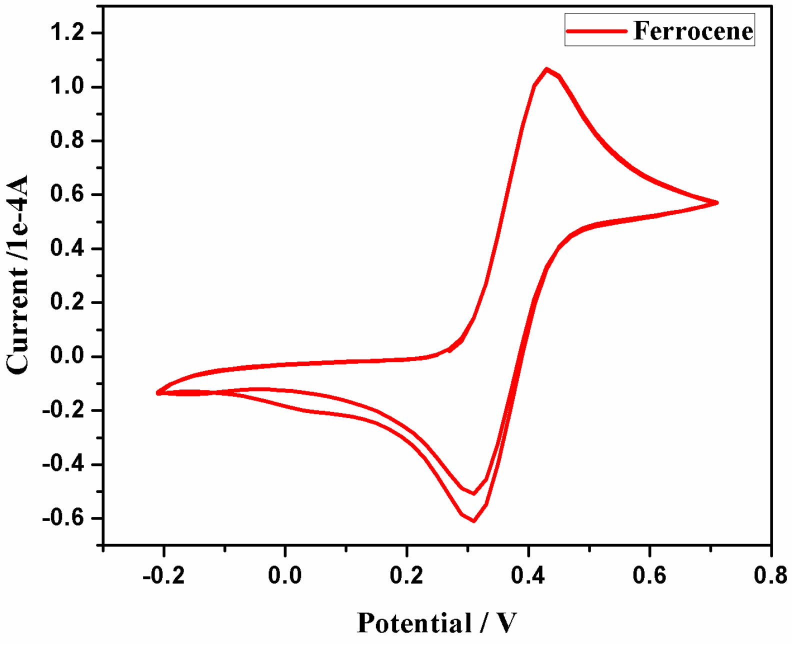

Figure S4. Cyclic voltammogram of Fc/Fc $^{+}$ couple as reference (E° (Fc/Fc $^{+}$) = 0.63 V vs. Ag/AgCl)

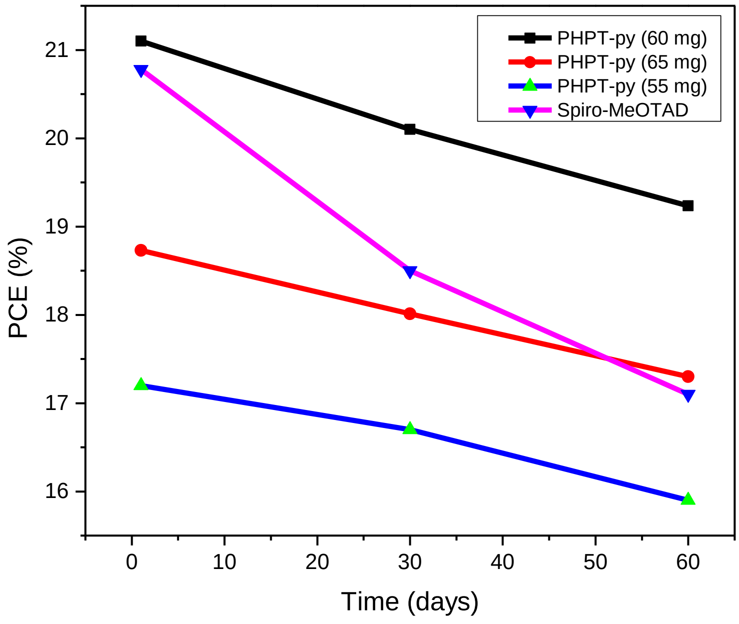

Figure S5. Stability of the perovskite films and devices long-term stability of PSCs for two months. These PSCs were measured and stored in glove box before and after *J–V* measurement.

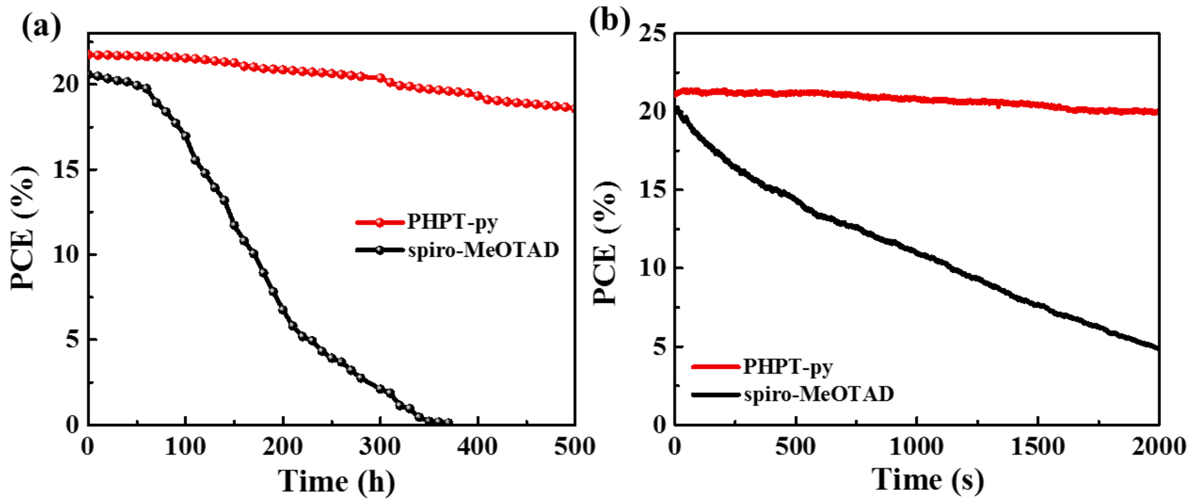

Figure S6. (a) The stability of perovskite solar cells under ambient environment of 55% relatively humidity without encapsulation at room temperature (b) Changes of PCE *vs.* illumination time of PSCs in presence of PHPT-py and spiro-MeOTAD as HTM obtained by tracking the maximum power point at 0.95 V.

Table S1: Comparison of cost estimation and photovoltaic performance among common HTMs in perovskite solar cells and PHPT-py HTM reported in this work.

HTM	Hole mobility ($cm^2V^{-1}S^{-1}$)	Cost/g ($)*	Perovskite Material	Dopants	PCE	V_{OC}	Ref
**PHPT-py**	7.6×10^{-5}	49	$MAPbI_3$	Rutin-AgNp, Li-TFSI, TBP	21.1	1.109	Our work
**Spiro-MeOTAD**	$10^{-3} – 10^{-4}$,	603	$MAPbI_3$	Li-TFSI, TBP	21.71	1.08	[3, 4]
**FDT**	------	60 [5]	$FAPbI_3/MAPbBr_3$	Li-TFSI, TBP, FK209	20.2	1.14	[5]
**P3HT**	<0.1, [6]	400–450	$MAPbI_3$	LiTFS, TBP, PMMA	15.3	1.02	[7]
**PTAA**	$10^{-2} – 10^{-3}$ [8]	2500–3000	$(FAPbI_3)_{1-x}(MAPbBr_3)_x$	Li-TFSI, TBP	20.2	1.06	[9]
**PEDOT:PSS**	$10^{-2} – 10^{-3}$ [10]	200–250	$MAPbI_3$	LiTFSI, TBP	18.1	1.1	[11]
**PDPPDBTE**	0.46 [12]	400	$MAPbI_3$	free	9.2	0.8	[13]
**DOR3T-TBDT**	0.26 [14]	350	$MAPbI_{3-X}Cl_X$	free	14.9	0.97	[14]

*The price is taken from Sigma Aldrich website (https://www.sigmaaldrich.com/germany.html)

FDT: 2,7-bis(4-methoxyphenyl)amino) spiro[cyclopenta (2,1-b:3,4-b)dithiophene-4,9-fluorene. **TBP**: 4-tert-butylpyridine, **Li-TFSI**: lithium bis(trifluoromethylsulphonyl) imide. **FK209**: Tris(2-(1H-pyrazol-1-yl)-4-tert-butylpyridine)-Tris(bis(trifluoromethylsulfonyl)imide). **PMMA**: poly(methyl methacrylate).

Table S2. UV-*vis* and electrochemical data for HTMs

HTM	$\lambda_{abs, max}$ [nm]	$\lambda_{em, max}$	HOMO (V)	E_{0-0} a) [eV]	E_{ox} b) (V)	E_{LUMO} c) [V vs NHE]
**Rutin-AgNP**	395	380	-4.97	3.14	0.10	-1.83
	390	365	-4.82	3.18	0.10	-1.64
**PHPT-py**	420	480	5.09	2.95	0.20	-2.14
	415	454	-4.18	2.99	0.20	-1.19
**Spiro-MeOTAD**	380	417	-5.12	3.01	0.13	-2.11
	378	413	-5.05	3.08	0.13	-1.97

a) Calculated from the intersection of the normalized absorption and emission spectra; b) The ground state oxidation potentials of the dyes were estimated under the following conditions: Pt as counter electrode and glass carbon working electrode. The electrolyte consisted of 0.2 m M dye and 0.05 M $[(Bu_4)N]PF_6$ in dichloromethane. The reference Ag/Ag^+ electrode was calibrated against an internal Fc/Fc^+ reference (E° (Fc/Fc^+) = 0.63 V vs. NHE) c) Estimated by subtracting $E_{0–0}$ from the oxidation potential.

References

[1] V. Niemi, P. Knuuttila, J.-E. Österholm, J. Korvola, *Polymer* **1992**, 33, 1559.

[2] S. Amou, O. Haba, K. Shirato, T. Hayakawa, M. Ueda, K. Takeuchi, M. Asai, *Journal of Polymer Science Part A: Polymer Chemistry* **1999**, 37, 1943.

[3] N. Ahn, D.-Y. Son, I.-H. Jang, S. M. Kang, M. Choi, N.-G. Park, *J. Am. Chem. Soc.* **2015**, 137, 8696.

[4] D. Bi, C. Yi, J. Luo, J.-D. Décoppet, F. Zhang, S. M. Zakeeruddin, X. Li, A. Hagfeldt, M. Grätzel, *Nature Energy* **2016**, 1, 16142.

[5] M. Saliba, S. Orlandi, T. Matsui, S. Aghazada, M. Cavazzini, J.-P. Correa-Baena, P. Gao, R. Scopelliti, E. Mosconi, K.-H. Dahmen, *Nature Energy* **2016**, 1, 15017.

[6] I. McCulloch, M. Heeney, C. Bailey, K. Genevicius, I. MacDonald, M. Shkunov, D. Sparrowe, S. Tierney, R. Wagner, W. Zhang, *Nature materials* **2006**, 5.

[7] S. N. Habisreutinger, T. Leijtens, G. E. Eperon, S. D. Stranks, R. J. Nicholas, H. J. Snaith, *Nano Lett.* **2014**, 14, 5561.

[8] J. H. Heo, S. H. Im, J. H. Noh, T. N. Mandal, C.-S. Lim, J. A. Chang, Y. H. Lee, H.-j. Kim, A. Sarkar, M. K. Nazeeruddin, *Nature photonics* **2013**, 7, 486.

[9] W. S. Yang, J. H. Noh, N. J. Jeon, Y. C. Kim, S. Ryu, J. Seo, S. I. Seok, *Science* **2015**, 348, 1234.

[10] S. Rutledge, A. Helmy, *J. Appl. Phys.* **2013**, 114, 133708.

[11] J. H. Heo, H. J. Han, D. Kim, T. K. Ahn, S. H. Im, *Energy & Environmental Science* **2015**, 8, 1602.

[12] T. K. An, I. Kang, H. j. Yun, H. Cha, J. Hwang, S. Park, J. Kim, Y. J. Kim, D. S. Chung, S. K. Kwon, *Adv. Mater.* **2013**, 25, 7003.

[13] Y. S. Kwon, J. Lim, H.-J. Yun, Y.-H. Kim, T. Park, *Energy & Environmental Science* **2014**, 7, 1454.

[14] Y. Liu, Q. Chen, H.-S. Duan, H. Zhou, Y. M. Yang, H. Chen, S. Luo, T.-B. Song, L. Dou, Z. Hong, *Journal of Materials Chemistry A* **2015**, 3, 11940.
